# Preoperative computerized tomography-guided blue dye localization for metastatic lymphadenopathy and peritoneal tumor implants during laparoscopic surgery: Two case reports

**DOI:** 10.1097/MD.0000000000016159

**Published:** 2019-06-21

**Authors:** Bi Li Yang, Kuo Chang Chen, Chin Chu Wu

**Affiliations:** Department of Diagnostic Radiology, Shin Kong Wu Ho-Su Memorial Hospital, Taipei, Taiwan.

**Keywords:** abdominal cavity, adult, preoperative period, sulfan blue, tomography

## Abstract

**Rationale:**

Computerized tomography (CT)-guided blue dye localization has been widely discussed for preoperative localization of pulmonary nodules. However, few studies have investigated this technique for intra-abdominal lesions. Although preoperative localization is not commonly required in laparotomy, it may assume importance with advancements in the field of laparoscopic surgery.

**Patient concerns:**

Herein, we report the cases of 2 patients diagnosed with colon cancer who underwent hemicolectomy with extended lymphadenectomy and subsequent chemotherapy.

**Diagnoses:**

Follow-up CT scans showed newly developed metastatic lymphadenopathy and peritoneal tumor implants.

**Interventions:**

Considering the difficulty in identification of and access to the target lesions during laparoscopic surgery, preoperative CT-guided blue dye localization was performed in both cases.

**Outcomes:**

All the target lesions were identified by the dye marker and removed successfully. The pathologic results revealed adenocarcinoma.

**Lessons:**

We established the following strategy for preoperative CT-guided dye localization of intra-abdominal lesions:

Intra-abdominal lesions that are hard to identify due to their size or morphology, and difficult to approach due to their location or surrounding structures, maybe the candidates for this procedure, especially in cases of laparoscopic surgery.

Operators should adjust their localization planning based on the surgery method, cutting path, and location of port sites. The target dye marker should be clearly visible in the presumed intra-operative field of view.

A second dye marker should be made to ensure surgical success when the target dye marker is obscured by the surrounding structures in the presumed intra-operative field of view.

## 1 Introduction

Computerized tomography (CT)-guided blue dye localization is a well-developed technique and is already being used in many clinical cases. It has been widely discussed for preoperative localization for wedge resection of pulmonary nodules.^[1–3]^ However, few studies have investigated this technique for intra-abdominal lesions,^[4]^ which may be because of the fact that most intra-abdominal lesions can be observed directly during the surgery. However, in cases where identification of a lesion is challenging or the target location is difficult to access, especially during laparoscopic surgery, preoperative localization may play an important role in ensuring surgical success. Herein, we report the cases of 2 patients who underwent CT-guided blue dye localization for metastatic lymphadenopathy and peritoneal tumor implants because of colon cancer before laparoscopic surgery. We discuss the advantages and disadvantages of using this technique in these 2 cases, which may help operators develop improved strategies for localization planning.

## 2 Case presentation

### 2.1 Case 1

**Figure 1:**
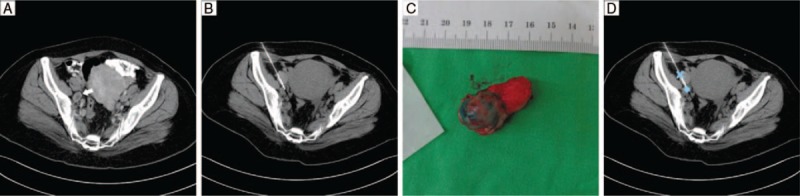
(A) Preoperative CT image in Case 1 showing a 13-mm lymphadenopathy (arrow) at the right internal iliac chain, posterior to the right psoas muscle. (B) CT-guided needle localization of the target lymph node. (C) Successful blue coloration of the gross specimen. (D) The blue dot represents the target lymph node. The blue cross represents the second dye marker that should have been made at the ventral surface of the right psoas muscle. CT = computerized tomography.

A 49-year-old woman diagnosed with ileocecal valve adenocarcinoma in October 2015 underwent right hemicolectomy with extended lymphadenectomy and subsequent chemotherapy. Pathologic analysis revealed American Joint Committee on Cancer (AJCC) 7th stage pT4aN2bM0. After 17 months of regular follow-up, CT imaging showed newly developed right internal iliac lymphadenopathy with a short axis diameter measuring up to 13 mm (Fig. 1A). Positron emission tomography (PET) revealed 18F-fluorodeoxyglucose uptake and nodal metastasis was strongly indicated. Considering that it would be difficult to identify and access the target lesion during laparoscopic surgery due to the overlying right psoas muscle, preoperative CT-guided blue dye localization was performed. After local anesthesia and percutaneous puncture with a 22-gauge percutaneous transhepatic cholangiography needle (Hakko, Japan) via an anterior approach, 0.5 mL of pure PATENT BLUE V (Guerbet, France) was injected into the target node (Fig. 1B). However, the surgeon could not recognize the target node via the laparoscopic ports in the supra-umbilical, infra-umbilical, and left lower abdomen regions because the dye marker was mostly hidden underneath the psoas muscle. After placing an additional port site in the right groin area, a well-defined node with dye marker was identified. The size, shape and adjacent anatomical landmarks of the node corresponded to our measurement in preoperative CT. There was no other suspicious lesion in the intraoperative field. The target node was resected uneventfully (Fig. 1C). The pathology result showed metastatic adenocarcinoma, representing stage IV status. After 5 days of hospitalization, the patient was referred to another hospital for further chemotherapy.

### 2.2 Case 2

**Figure 2:**
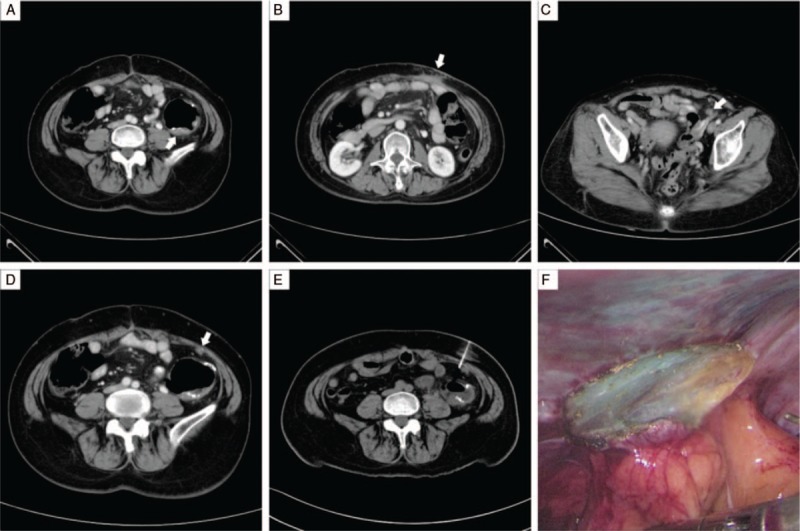
(A) Preoperative CT scan in Case 2 showing local recurrence at the anastomotic site (arrow). (B–D) Three sites of tumor implants involving the anterior abdominal wall and left lower peritoneum (arrows). (E) CT-guided needle localization of the target nodule in the left lower abdomen. (F) Endoscopic image showing successful blue coloration of the target nodule. CT = computerized tomography.

A 55-year-old woman diagnosed with descending colon adenocarcinoma in January 2017 underwent left hemicolectomy with extended lymphadenectomy and subsequent chemotherapy. Pathologic analysis revealed AJCC 7th stage pT4aN1aM0. After 8 months of regular follow-up, the carcinoembryonic antigen level increased from 0.5 ng/mL to 5.9 ng/mL, and CT scan demonstrated suspicious local recurrence (Fig. 2A) and 3 sites of peritoneal tumor implants with surrounding adhesion (Fig. 2B–D). The subsequent PET scan also suggested the impression. Believing that it would be difficult to identify the smallest tumor implant (5 mm, Fig. 2D) during laparoscopic surgery, preoperative CT-guided blue dye localization with 0.3 mL of pure PATENT BLUE V was performed (Fig. 2E). The target lesion was clearly identified with the dye marker during the surgery (Fig. 2F). All tumor implants corresponded to preoperative CT in size and location were removed, along with the local recurrence. The pathology results all revealed adenocarcinoma. Her disease remained stable during the first 5 months of chemotherapy with folinic acid-fluorouracil-irinotecan (FOLFIRI) regimen. Small new growing implants were found in CT at 8-month follow-up. The treatment plan was changed to chemotherapy with FOLFIRI regimen plus targeted therapy with bevacizumab. Nevertheless, the treatment effect was limited and the latest CT showed disease progression in January 2019.

## 3 Discussion

Studies have indicated that CT-guided blue dye localization for pulmonary nodules exhibits a high success rate (94%–98%),^[2,3,5]^ and CT-guided biopsy and drainage of the abdominal cavity have been widely applied. It can be inferred that the success rate of CT-guided blue dye localization for intra-abdominal lesions is considerably high. Although preoperative localization is not commonly required in laparotomy, this technique may assume importance with the developments in the field of laparoscopic surgery. In the cases described in this report, CT-guided blue dye localization was applied successfully for better identification of the target lesion during laparoscopic surgery.

In Case 1, preoperative blue dye localization was performed because of difficulty in accessing the target lesion due to the overlying psoas muscle. Although the procedure was successful, the surgeon did not identify the lesion via the laparoscopic ports at the beginning of the surgery because the dye marker was mostly obscured underneath the muscle. To avoid this, precise surgical planning (including cutting path and port site locations) and the presumed intra-operative field of view should be put into consideration when the operator determines the exact extent of the dye marker. Furthermore, a second dye marker is required when the target dye marker is obscured by the surrounding structures. In this case, the second dye marker should have been made at the ventral surface of the psoas muscle so that the surgeon could have noticed the to-be-cut level of the target lesion at first sight (Fig. 1D).

In Case 2, preoperative assessment revealed multiple target sites with surrounding adhesion. Hence, blue dye localization for the smallest tumor implant was performed because of the surgical complexity. Although the target tumor nodule itself was not difficult to access, preoperative localization shortened the duration of laparoscopic surgery and resulted in a favorable outcome by facilitating identification of the subtle lesion.

Technically, CT-guided blue dye localization is a safe and efficient procedure with a high success rate. Before the procedure, a detailed history taking should be performed to know whether the patient has had specific allergies, severe claustrophobia, or severe coagulopathy. When performing intra-abdominal localization, a safe path for puncture should be established to avoid hollow organs and large vessels. Patients should change their posture, take oral diluted iodine contrast medium, or receive intravenous iodine contrast medium injection to avoid non-target puncture, if necessary. According to published literature on the localization of pulmonary nodules and our experiences with the localization of both pulmonary and intra-abdominal lesions, 0.3 to 0.5 mL of pure blue dye is adequate for a lesion measuring <10 mm.^[2,3,5,6]^

Kleedehn et al retrospectively reviewed 107 cases of lung nodules subjected to wire or blue dye localization, and no significant differences between these 2 localization methods were observed in terms of the success rate and complications. Although wire dislodgement occurred in 13% of the cases, no influence on the surgical outcome was noted.^[5]^ In addition, wire causes pleural or peritoneal irritation, whereas dye localization has no such disadvantages.^[7]^

In our facility, patients undergo the surgery within 3 hours after completion of wire localization in case of dislodgement. On the other hand, according to our experience, the injected dye marker may last up to 24 hours in vivo and still offer the surgeon precise identification of the target lesion. Therefore, dye localization offers greater flexibility with respect to scheduling of CT examination rooms and operating rooms.

Some studies have shown that blue dye causes allergies.^[8–11]^ Montgomery et al reported that among 2392 patients who underwent sentinel lymph node mapping for breast cancer, the incidence of allergy to isosulfan blue dye and hypotensive reactions was 1.6% and 0.5%, respectively.^[11]^

We have established the following strategy for preoperative CT-guided dye localization of intra-abdominal lesions

Intra-abdominal lesions that are hard to identify due to their size or morphology, and difficult to approach due to their location or surrounding structures, maybe the candidates for this procedure, especially during laparoscopic surgery.

Operators should modify their localization planning based on the surgical method, cutting path, and location of port sites. The target dye marker should be clearly visible in the presumed intra-operative field of view.

A second dye marker should be made to ensure surgical success when the target dye marker is obscured by the surrounding structures in the presumed intra-operative field of view.

In summary, this article highlights the clinical importance of preoperative CT-guided dye localization for select cases during laparoscopic surgery. Further experience is needed to advance the protocol for patient selection and improve the strategies for localization planning.

## Author contributions

**Data curation:** Bi Li Yang.

**Project administration:** Bi Li Yang.

**Supervision:** Chin Chu Wu.

**Writing – original draft:** Bi Li Yang.

**Writing – review and editing:** Bi Li Yang, Kuo Chang Chen, Chin Chu Wu.
